# Small pushes, big impact: nudges that transform stewardship practice, results of a quasi-experimental study using clinical vignettes

**DOI:** 10.1017/ash.2026.10389

**Published:** 2026-05-05

**Authors:** Stephanie J. Harding, Nick M. Harris, Christina M. Brummett

**Affiliations:** 1 Pharmacy, Wesley Medical Center, Wichita, USA; 2 Laboratory, Wesley Medical Center, Wichita, USA

## Background

Antimicrobial stewardship programs (ASPs) are designed to optimize antimicrobial use. Traditional stewardship models are often labor intensive and rely on audit and feedback.^1–3^ Diagnostic stewardship (DS) involves adjusting diagnostic tests to prompt optimal patient care. Although DS may be labor intensive upfront for electronic medical record builds, it often requires less labor over time. DS initiatives often can retain their effect long after implementation and may withstand some external influences.^4–10^

ASPs may leverage DS interventions to maximize the reach of their program. In 2019, Wesley Healthcare’s ASP wanted to expand its DS efforts. Providers at this tertiary community teaching hospital were surveyed on their interpretation of common microbiology results to evaluate areas of perceived need. Updates were made to optimize microbiology results based on survey results, contextual inquiries, literature reviews, and interviews with provider groups. A repeat survey was completed in 2024. The purpose of this study was to evaluate if the changes positively impacted clinical decisions based on these results.

## Methods

This was a quasi-experimental study evaluating the impact of different verbiages on common microbiology results. The study was completed by recruiting physicians, advanced practice professionals, pharmacists, and residents to participate in a pre-/post-study composed of six multichoice clinical vignettes utilizing the current presentation of various microbiology results.

The vignettes included questions on antibiotic initiation and/or de-escalation through interpretation of results for various pathogens and minimum inhibitory concentrations. Our primary outcome was rate of appropriate responses between the pre- and postupdate surveys. Chi-squared analysis was used to calculate differences in the percentage of appropriate survey responses between the two groups.

## Results

Sixty-four respondents completed the survey in 2019 and 52 in 2024. Most common roles differed between the surveys. Medical residents were most common in the preupdate survey (44%) and attending physicians were most common in the postupdate survey (27%). Internal medicine and hospitalists were the two most common specialties in both years.

The survey included two clinical vignettes with detailed guidance added prior to 2019. *C. difficile* had guidance to aid in the differentiation of colonized versus infected patients. *S. pneumoniae* urine antigen results displayed a comment regarding the possibility of false positives postpneumococcal vaccination. Both showed sustained high rates of appropriate responses between surveys [*C. difficile* (Pre: 92%, Post: 98%) and *S. pneumoniae* urine antigen (Pre: 95%, Post: 94%)] (Table 1).

The two vignettes without updated guidance between surveys showed no significant difference in appropriate response rates. Group C *Streptococcus* results without specific susceptibility information led to low provider comfort in de-escalating antibiotics (Pre: 72%, Post: 81%). One question asked providers to narrow antibiotics based on MIC results; only annual education was provided with no updates to presentation. Survey responses showed no statistical difference between years (Pre: 72%, Post: 77%).

Vignettes with detailed microbiologic guidance added between surveys showed improved incidence of appropriate antibiotic management. In 2019, methicillin susceptible S*taphylococcus aureus* (MSSA) blood culture results utilized polymerase chain reaction (PCR) technology but were presented as “presumptive MSSA.” By 2024, results were modified to state “MSSA by PCR.” A significant increase was seen in providers selecting antibiotic de-escalation (Pre: 58%, Post: 92%, *P* ≤ .0001). *H. influenzae* results were updated to include “Beta lactamase not produced: ampicillin or amoxicillin are the drugs of choice” on results without resistance identified versus “Beta lactamase not produced” previously. Antibiotic de-escalation on this vignette also increased 86% pre and 98% post (*P* ≤ .02).

## Discussion

In this study, the addition of detailed actionable guidance was associated with higher rates of antibiotic optimization in the clinical vignettes. One important aspect to note was that verbiage may be vital to the impact of a diagnostic intervention. For example, the use of “presumptive” on blood cultures results was associated with lower rates of de-escalation. When this was modified to “by PCR,” antibiotic de-escalation increased significantly.

Education alone did not seem to improve appropriate management as shown on interpretation of MIC. Appropriate response rate did not significantly differ and MICs were frequently misinterpreted as being able to compare activity between antibiotics. Further research on the comparison between education versus updated detailed result presentation may be beneficial.

There are several possible confounders for this study. In addition to the selection/sampling, response, and confirmation biases common in survey-based research, the characteristics of our survey responders differed in several ways between our pre- and postupdate survey. More medical residents responded to the presurvey, while more advanced practice practitioners responded to the postsurvey. Our institution also welcomed a new, large, medical group composed of critical care intensivists attending physicians and advance practice practitioners as the admitting team for our intensive care unit patients between the two surveys. These differences in survey respondent training and specialty may have influenced the likelihood of selecting an appropriate response. Lastly, clinical vignettes were chosen as a surrogate to real-world antimicrobial de-escalation rates to highlight antimicrobial choice from microbiology result interpretation alone without external confounders seen in hospital de-escalation rates.

## Conclusion

This study emphasizes the importance of clear and detailed reporting of microbiology results. ASPs should evaluate how lab results are reported and interpreted and should work inter-professionally to ensure the verbiage used leads to optimal nudging.

**Table 1.**
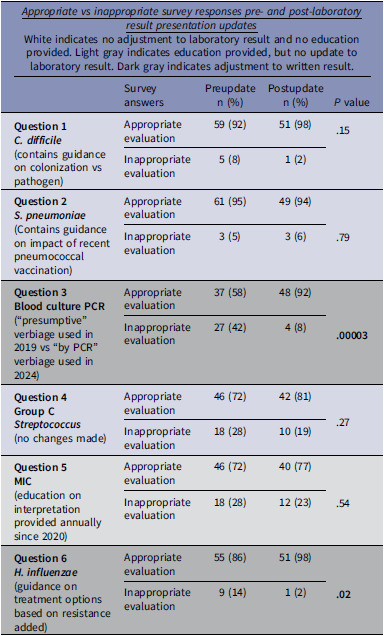
Survey results pre-/post-laboratory result presentation updates

## References

[1] CDC. Core elements of hospital antibiotic stewardship programs. Atlanta, GA: US Department of Health and Human Services, CDC. 2019. https://www.cdc.gov/antibiotic-use/core-elements/hospital.html. Accessed October 13, 2023.

[2] Jernigan JA . Multidrug-resistant bacterial infections in U.S. hospitalized patients, 2012–2017. N Engl J Med 2020;382:1309–1319. 32242356 10.1056/NEJMoa1914433 PMC10961699

[3] CDC. COVID-19: U.S. Impact on Antimicrobial Resistance, Special Report 2022. Atlanta, GA: .S. Department of Health and Human Services, CDC. 2022.

[4] Claeys KC , Johnson MD. Leveraging diagnostic stewardship within antimicrobial stewardship programmes. Drugs Context 2023;12:2022-9-5. 10.7573/dic.2022-9-5 PMC9949764 36843619

[5] Morency-Potvin P , Schwartz DN , Weinstein RA. Antimicrobial stewardship: how the microbiology laboratory can right the ship. Clin Microbiol Rev 2016;30:381–407. 27974411 10.1128/CMR.00066-16 PMC5217798

[6] Donner LM , Campbell WS , Lyden E , Van Schooneveld TC. Assessment of rapid-blood-culture-identification result interpretation and antibiotic prescribing practices. J Clin Microbiol 2017;55:1496–1507. 28250000 10.1128/JCM.02395-16 PMC5405267

[7] Patel R , Fang FC. Diagnostic stewardship: opportunity for a laboratory-infectious diseases partnership. Clin Infect Dis 2018;67:799–801. doi: 10.1093/cid/ciy077. 29547995 PMC6093996

[8] Morgan DJ , Malani P , Diekema DJ. Restrictive reporting of selected antimicrobial susceptibilities influences clinical prescribing. J Infect Public Health 2015;8:234–241. doi: 10.1016/j.jiph.2014.09.004. 25466592

[9] Morgan DJ , Malani P , Diekema DJ. Diagnostic stewardship-leveraging the laboratory to improve antimicrobial use. JAMA 2017;318:607–608. doi: 10.1001/jama.2017.8531. 28759678

[10] Torres C , Lyden E , Gillett G , Rupp ME , Van Schooneveld TC. Dropping the urine culture: sustained CAUTI reduction using a UTI order panel. Infect Control Hosp Epidemiol 2025;46:1–7. doi: 10.1017/ice.2025.2. PMC12015623 39943701

